# Alcohol, Excitotoxicity and Adult Brain Damage: An Experimentally Unproven Chain-of-Events

**DOI:** 10.3389/fnmol.2016.00008

**Published:** 2016-02-10

**Authors:** Michael A. Collins, Edward J. Neafsey

**Affiliations:** Department of Molecular Pharmacology and Therapeutics, Stritch School of Medicine, Loyola University ChicagoMaywood, IL, USA

**Keywords:** ethanol, glutamate, NMDA receptor, brain damage, oxidative stress

It has been claimed over the years—most recently in this journal and several others—that neuronal degeneration in adult brain arising during chronic alcohol exposure is, or is likely to be, *via* “excitotoxicity” (Chandrasekar, 2013; Mehta et al., 2013; Crews and Vetreno, 2014). A purpose of this opinion piece is to reinforce a view, noted earlier (Collins and Neafsey, 2012), that an excitotoxic mechanism underlying alcoholic neurodamage in the mature brain has never been experimentally proven *in vivo*. Indeed, pharmacological results in neuronal degeneration studies with repetitively intoxicated (binged) adult rats indicate that the excitotoxic route is insignificant—more about this later.

Discovered over 4½ decades ago (Olney, 1969), excitotoxicity relates principally to a deleterious property of the brain's major excitatory neurotransmitter, L-glutamate. When hyper-elevated extracellularly for a variety of reasons, glutamate can overstimulate neuronal N-methyl-D-aspartate (NMDA) receptors, causing cellular degeneration; sometimes increased receptor content could also be a factor. Specifically, excessively stimulated NMDA receptors—being calcium channels—facilitate the cytosolic accumulation of calcium ions, and elevated intraneuronal calcium triggers neuropathological events or associated accomplices that include, among others, increased protease, nuclease and phospholipase activities, oxygen- and nitrogen-free radicals (oxidative/nitrosative stress), membrane lipid peroxidation, and perhaps reductions in antioxidant defenses (Pina-Crespo et al., 2014). The ensuing neuronal degeneration can be apoptotic, necrotic, or a continuum of both. Excitotoxicity is considered a chief mechanism for brain damage in acquired insults/models such as stroke, trauma, status epilepticus, hypoglycemia, and hyperglycemia. It also may have a role in the chronic neurotoxicity of toxicants such as methyl mercury and trimethyltin, and of severe vitamin deficiencies (notably, thiamine). In any event, the *sine qua non* proof of excitotoxicity-mediated neuronal degeneration in these and other experimental conditions is pharmacological—blockade of glutamatergic (especially NMDA) receptors and calcium elevations by effective antagonists should significantly suppress or abrogate neuronal demise.

The original proposals that excitotoxicity causes chronic alcohol-dependent brain damage were based on evidence entirely from brain/neuronal cultures or cell lines, and not in *in vivo* adult models (Lovinger, 1993; Tsai et al., 1995). Numerous culture-based reports have appeared since those first experiments (e.g., Hendricson et al., 2007)—most recently, studies with neonatal or early adolescent-age hippocampal slice cultures treated with high alcohol for a week or more, followed by withdrawal, in which NMDA receptor antagonism reduces neurodegeneration (Stepanyan et al., 2008; Reynolds et al., 2015). However, these and other brain cultures, being perinatal and/or developmental and (in the case of dispersed cultures or cell lines) often lacking glial and cerebrovascular components, may well be insufficient or even inappropriate models for alcohol's neurotoxic mechanisms in the intact adult brain. Among dissimilarities in outcomes, neuronal apoptosis can be prominent in binge alcohol-treated rodent brain cultures, but is largely absent in brain regions exhibiting neuronal degeneration in a repetitive binge-intoxicated adult rat model to be described subsequently (Obernier et al., 2002). Also, changes in systemic proinflammatory agents such as certain cytokines that are linked to neuronal degeneration are of course not present with *in vitro* models.

Proof of excitotoxicity-associated neuronal degeneration in adult brain from alcohol requires *in vivo* studies employing NMDA receptor antagonists, but such studies have been rare. For discussion purposes, two general approaches have been used to study chronic alcohol-induced brain damage. In the first, voluntary or semi-voluntary oral intake of alcohol in water or liquid diets for weeks or sometimes months, the maximal blood alcohol concentration (BAC) reached is considered experimentally moderate, generally encompassing 80–150 mg/dl; among variations, “drinking-in-the-dark” or regulated episodic access can achieve higher values. Since the seminal reports in the 1980's by the late Don Walker and collaborators, these adult models are documented to incur brain neuronal loss from chronic alcohol ingestion.

Amid many studies using these drinking models or their variations, none appears to have examined in detail the quantitative impact of NMDA receptor antagonism on alcohol-dependent brain neuronal degeneration and/or loss. We could locate only a few chronic alcohol ingestion studies that used NMDA receptor antagonists, and these provide insufficient information on excitotoxicity *per se*. In the first of these, the influence of weeks of alcohol+liquid diet intake on brain injury arising from ischemia/reperfusion employed adult rats co-treated with memantine, a relatively specific NMDA receptor antagonist; however, memantine's effects on the neuronal loss or damage expected from the alcohol intake alone were unspecified (Zhao et al., 2011). An earlier study, albeit with rats of late adolescent-age, used alcohol+liquid diet ingestion for 2 weeks (maximal BAC, 126 mg/dl) to assess the effect of NMDA receptor antagonism on brain oxidative stress indicators (Bondy and Guo, 1996). Unsurprisingly, alcohol intake significantly increased brain oxidative stress as reflected in regional declines of antioxidants glutathione and superoxide dismutase, but co-administration of MK-801, another potent NMDA receptor antagonist, failed to inhibit the decreases. Since MK-801 should have interrupted oxidative stress if derived from NMDA receptor-linked excitotoxicity, the authors opined that “the pro-oxidant properties of ethanol may thus act independently of its actions upon the NMDA receptor.”

The second basic approach to realizing neuronal degeneration in adult animals has been involuntary alcohol intoxication, either chronic (>8 days) or subchronic (~4 days), principally *via* intragastric (i.g.) intubation, vapor inhalation, or intraperitoneal (i.p.) injection. Again, different doses, schedules and durations have been employed for alcohol delivery. Often the maximal BAC in rodents given the typically medium to high doses (3–5 g alcohol/kg/d i.g. or i.p.) is similar to those in severe chronic alcoholics—in the approximate range of 300–450+ mg/dl. Such concentrations, although seemingly high, match those seen in toxicology assays in active, severe alcoholics. If alcohol is provided to adult rats over 4 days via thrice-daily intragastric binges (developed to study dependence-related seizures, Majchrowicz, 1975), neuronal degeneration that, similar to human alcoholism, is prominent in hippocampus and temporal cortex (Collins et al., 1996).

Anticipating excitotoxicity in this binge model, we first tested MK-801's effects and, as noted in an initial abstract, found no observable neuroprotection in adult male rats (Neafsey et al., 1989). Expansion of the studies led to full reports detailing the lack of significant prevention of the neurodamage by MK-801 in the 4-day model (Corso et al., 1998) as well as in a modified once-a-day gavage technique over 7–10 days, which afforded the regional degeneration with considerably lower maximal BACs (Collins et al., 1998). Also not neuroprotective in the 4-day model were antagonists of glutamatergic AMPA receptors (DNQX) and neuronal calcium channels (nimodipine), and inhibitors of nitric oxide synthases—pathways connected to the excitotoxic mechanism (Zou et al., 1996; Corso et al., 1998). Subsequently, lack of evidence for excitotoxicity in the Majcrowicz binge model was verified in R. Eskay's laboratory at NIAAA using memantine as well as nimodipine (Hamelink et al., 2005). However, brain oxidative stress is critical to the observed neuropathology, since antioxidants are effective protectants (Hamelink et al., 2005; Crews et al., 2006). Thus, as with the liquid diet experiments by Bondy and Guo mentioned above, the ostensible free radical elevations do not appear to be driven by an excitotoxic mechanism.

We were unable to find other studies of involuntary intoxication in a mammalian model that specifically examine neurotoxicity with NMDA receptor antagonists. However, alcohol vapor-exposed adult drosophila sustained degeneration of olfactory neurons that was termed excitotoxic due to blockade by lithium (French and Heberlein, 2009). Among its many targets, lithium inhibits glycogen synthase kinase-3 (GSK3), which some reports associate with potentiation of excitotoxicity. However, it would have been more compelling in this non-mammalian model if blockade had been accomplished with an NMDA receptor antagonist, as there are a number of explanations for lithium's neuroprotective results besides excitotoxic inhibition (and GSK3 can mediate cell death by routes other than excitotoxicity). Prior to this drosophila study, lithium neuroprotection against alcohol had been reported only in primary neuronal cell cultures or developing brain.

Continuing the subject of developing CNS, alcohol-induced neuronal pathology might well involve excitotoxic mechanisms in fetal or perinatal brain, as indicated by the rodent brain culture studies cited earlier; *in vivo* verification is provided with 6-day old rat pups given acute alcohol binges, in which MK801 treatment significantly attenuated later neurobehavioral impairments (Thomas et al., 2002 and earlier reports from the Riley laboratory). Parenthetically, neuronal protection is unreported in the above citation, but is mentioned in a concurrent RSA abstract with another NMDA receptor antagonist. Furthermore, many investigations have detailed numerous brain glutamate/NMDA receptor-related changes and neurobehaviors in adult rodents during prolonged alcohol exposure (Tsai and Coyle, 1998)—e.g., alterations *in vivo* in glutamatergic neurotransmission, NMDA receptor densities, synaptic and neuroadaptive phenomena such as pruning, ionic disturbances or flux, neuroplasticity, and withdrawal-associated seizures, among others. Moreover, NMDA receptor antagonism can significantly perturb many of these alcohol-induced brain effects (Nelson et al., 2005; Holmes et al., 2012; Navarro and Mandyam, 2015). Thus, a functional hyperglutamatergic state is associated with chronic alcohol in several regions of adult brain. We further acknowledge that alcohol treatments might potentiate excitotoxic mechanisms promoted by other insults such as brain ischemia/reperfusion (Zhao et al., 2011); additionally, brain damage in the Wernicke-Korsakoff syndrome in alcoholics might include a prominent excitotoxic component, since neurodamage from experimental thiamine deficiency, which primarily underlies Wernicke's, can be antagonized by NMDA receptor blockade.

However, with respect to adult brain neuronal degeneration from short-term daily binges, pharmacological findings repeatedly fail to support excitotoxicity in the mechanistic chain-of-events. In contrast, brain damage in such models (with both adult rats and mice) is linked to oxidative stress-linked mechanisms apparently encompassing pro-inflammatory/neuroimmune cytokine and lipid pathways (Crews and Vetreno, 2014; Tajuddin et al., 2014). Admittedly, these short term adult animal procedures might be viewed as inadequate models for alcoholic brain damage arising from decades of dependence—but they are still closer to the human condition than neonatal brain cultures. If excitotoxicity is critical to neurodamage in adult models of prolonged daily alcohol intake that mirror chronic alcoholism, experimental verification is needed with receptor antagonists or perhaps receptor silencing using RNA interference. Until convincingly demonstrated in these models, excitotoxicity in alcohol-induced adult brain damage remains solely speculative and unproven.

## Author contributions

MC and EN were both involved in conceiving and drafting this opinion piece, and providing final approval of the version to be published. They further do agree to be accountable for all aspects of it related to its accuracy or integrity.

### Conflict of interest statement

The authors declare that the research was conducted in the absence of any commercial or financial relationships that could be construed as a potential conflict of interest.
